# Traditional Chinese medicine for sjögren’s syndrome: a national survey of attitudes and perceptions among Chinese patients and rheumatologists

**DOI:** 10.1186/s12906-024-04591-2

**Published:** 2024-07-30

**Authors:** Ruihua Liu, Zilin Guo, Haodong Xu, Jing He, Bei Wang, Yuebo Jin, Ziying Zhao, Xiaopo Tang, Xinyao Zhou

**Affiliations:** 1 Guang’anmen Hospital, Chinese Academy of Traditional Chinese Medicine, Beijing, China; 2 Beijing University of Traditional Chinese Medicine, Beijing, China; 3 Suzhou TCM Hospital Affiliated to Nanjing University of Chinese Medicine, Jiangsu, China; 4 Peking University People’s Hospital, Beijing, China; 5 Beijing Hospital of Traditional Chinese Medicine, Capital Medical University, Beijing, China

**Keywords:** Sjögren’s syndrome, Traditional Chinese medicine, Patients with Sjögren’s syndrome, Rheumatologists, Attitude

## Abstract

**Background:**

This study explored similarities and differences among Chinese patients and rheumatologists in their attitudes towards and perceptions of traditional Chinese medicine (TCM) for Sjögren’s syndrome (SS), including analyzing factors that influenced their decision making.

**Methods:**

An anonymous questionnaire was used to conduct a multicenter survey among patients with SS at three tertiary care medical centers in Beijing and among rheumatology clinicians at several hospitals across China. Results were analyzed using descriptive statistics.

**Results:**

There were 942 valid questionnaires from patients from 31 provinces and cities in China, with a male-to-female ratio of approximately 1:14, a mean age of 48.81 years, and a median disease duration of 7 (4, 10) years. There were 320 valid questionnaires from rheumatologists, covering 30 provinces and cities in China, with a male-to-female ratio of approximately 0.87:1, a mean age of 48 years, and a median work duration of 10.5 (6, 15) years. The rheumatologists treated a median of 15 (11, 50) SS cases per month, and the median proportion of SS to all rheumatic diseases was 6.66% (6–10%). Many patients believed TCM could cure the root of the disease, and the most expected TCM therapies were TCM patent prescriptions and medicinal teas. Conversely, rheumatologists placed high value on the efficacy of TCM, and most commonly prescribed Chinese herbal decoctions. Most doctor-patient groups were positive about TCM treatment, citing the low side effects as the major advantage. Regression analysis showed that for patients over 40 years old with a course of disease > 4 years, the probability of using TCM has increased by 1–6 times; the probability of recommending TCM in clinical work of doctors who have worked for more than 15 years, TCM and integrated traditional Chinese and western medicine has increased 1–2 times.

**Conclusions:**

TCM has become widely accepted and earned attention from doctor-patient groups, especially among older patients and experienced rheumatologists. However, negative prejudices and absence of accurate information about TCM treatments and SS itself require improvement. The contradiction between TCM dosage form and efficacy is a major problem, and patient demand for convenient and efficient TCM patent preparations suggests future work should focus on developing TCM patent preparations with clear compositions and mechanisms.

## Introduction

Sjögren’s syndrome (SS) is a chronic autoimmune disease that predominantly involves the exocrine glands. The common clinical symptoms of SS include salivary gland dysfunction, lymphocyte infiltration, and autoantibodies [1]. The prevalence of SS ranges from 0.01% to 3% [2] and is increasing annually. Patients with SS are prone to concomitant complications and multisystem damage, which results in a greater need for clinical treatment [3]. The etiology and pathogenesis of SS remain unknown, limiting the development of safe and efficient clinical treatment protocols [4]. China has two medical systems, Chinese medicine and Western medicine, and treatments from both systems are readily available. While the mechanisms behind the efficacy of Western medical treatments for SS are relatively clear, these treatments have a poor safety profile and an inadequate ability to control local symptoms and disease progression [5, 6]. Furthermore, the effects of widely used artificial fluid replacement therapy to relieve dry mouth and dry eyes are not ideal [7]. Traditional Chinese medicine (TCM) involves deeply rooted holistic concepts and syndrome differentiation for treatment, and some patients and rheumatologists have turned to TCM treatments as complementary or alternative therapies for SS.

Previous surveys reported that the use of TCM in the treatment of SS is as high as 72.20% [8]. Many studies have shown that TCM plays a role in improving dryness symptoms and delaying disease progression in patients with SS [9–13], while also helping to relieve their anxiety and depression [14, 15]. In addition, TCM treatments have a good safety profile [16–18]. However, it was observed that some patients in China stop their routine treatments and follow-up owing to negative prejudices against using TCM, which creates difficulties for disease control. Some patients also use TCM at an inappropriate time, and consequently, the expected efficacy is not achieved. Moreover, rheumatologists have different levels of understanding and attitudes towards TCM for SS, which may lead to patient confusion. Clarity on all these issues is crucial for standardized disease management, patient education, clinical research on TCM, and application guidelines. In this study, a survey was performed at several hospitals across China to understand the application and demand of TCM by patients with SS, discern the attitudes and understanding of Chinese and Western rheumatologists towards TCM for SS, and discover the differences and deviations between patient and rheumatologist perceptions of TCM.

## Data and methods

### Sources of survey respondents

Questionnaires were distributed to patients with SS managed at the rheumatology departments of Guang’anmen Hospital of the Chinese Academy of Traditional Chinese Medicine, Peking University People’s Hospital, and the Beijing Hospital of Traditional Chinese Medicine from January 2022 to June 2022. Patient inclusion criteria must also meet the following conditions: (1) met the 2016 American College of Rheumatology (ACR)/The European League Against Rheumatism (EULAR) classification criteria for SS [19]; (2) not younger than 18 years old; (3) consent to accept the questionnaire survey. Rheumatologists inclusion criteria must also meet the following conditions: (1) engaged in rheumatism immunization professional of traditional Chinese medicine, western medicine and integrated Chinese and western medicine doctors; (2) consent to accept the questionnaire survey.

### Questionnaire and sampling size

This study adopts an anonymous questionnaire survey format. The development of the questionnaire refers to the high-quality survey literature in recent years [20–22], which was completed by rheumatologists at the rheumatology departments of Guang’anmen Hospital of the Chinese Academy of Traditional Chinese Medicine, Peking University People’s Hospital and the Beijing Hospital of Traditional Chinese Medicine who were ranked as associate chief rheumatologist and above. The questionnaire was divided into three parts. The first part recorded basic and participant-specific information. Patients were asked to report their region, gender, age, education, marital status, type of work, and annual family income. Rheumatologists were asked to report their gender, age, education, professional category, title, work unit level, and years of work. The second part of the survey described the patient’s illness or treatment regimen. The patient survey included the duration of illness, age of onset, first hospital visit, chief complaint, and sleep quality. The rheumatologist survey included the number and percentage of SS patients seen per month, the nature and frequency of condition assessment, and follow-up visits. The third part of the survey asked the participants about their perceptions and attitudes toward TCM therapies, including the role of TCM, the degree of acceptance or treatment recommendations, and the advantages and shortcomings of expected or recommended therapies. The full survey took 5–8 min to complete.

The literature [23] showed that the minimum sample size of the questionnaire was 5–10 times the number of set questions. In this study, there were 28 questions from patients and 23 questions from doctors, so the number of patients and doctors included in this study reached the minimum sampling size.

### Survey methodology

We conducted a combination of online and offline survey, and issued QR codes to patients and doctors who met the inclusion criteria. They could complete the questionnaire with their mobile phones. Before the formal investigation, 5 rheumatologists and 5 SS patients were invited to complete the questionnaire. After one week, the order of the questionnaire questions was disrupted, and these 5 rheumatologists and 5 patients were invited again to complete the questionnaire. The results of these two pre surveys were consistent. Some of the questionnaires had obvious logical errors, After we refer to the other high-quality survey literature [20–22], we decided to eliminate after discussion by two rheumatologists.

### Statistical methods

Statistical analysis was performed with SPSS 20.0 software (free version, IBM, Armonk, NY, USA). Data were described by number of cases, composition ratio, and 95% confidence interval (CI). Dichotomous logistic regression was used for multivariable analysis, with a two-sided *P* < 0.05 indicating a statistically significant difference. The rank sum test was used for ordered multicategorical data, and chi-square tests were used for unordered multicategorical data, with *P* < 0.05 indicating a statistically significant difference. GraphPad Prism 8.0.2 software (La Jolla, CA, USA) was used for plotting.

## Results

### General information

A total of 942 patients were included in the study, covering 31 provinces and cities across China. Sixty-one patients (6.48%) were male, and 881 patients (93.52%) were female, with a male-to-female ratio of approximately 1:14. The mean age was 48.81 years, and the median disease duration was 7 (4, 10) years. Medical insurance was reported by 884 patients (93.84%), while 58 had no medical insurance (6.16%). Family annual income was primarily less than 100,000 yuan (681, 72.29%), while 23.78% (224) patients earned 100,00–300,000 yuan. Patient knowledge about SS predominantly came from hospital doctors (657, 69.75%) and TV networks (223, 23.67%). Most patients attached more importance to the disease and hoped not to progress (493, 52.34%), while more than 1/3 of patients hoped for a complete cure (367, 38.96%).

A total of 320 rheumatologists were included in the study, covering 30 provinces and cities nationwide. The male-to-female ratio was approximately 0.87:1, with an average age of 48 years and a median work experience of 10.5 (6, 15) years. Titles were predominantly chief rheumatologists (118, 36.88%), followed by attending rheumatologists (91, 28.44%) and associate chief rheumatologists (81, 25.31%). More than 50% of the specialty categories were Chinese medicine (177, 55.31%), followed by combined Chinese and Western medicine (74, 23.13%) and Western medicine (69, 21.56%). The highest education level of the respondents was most commonly a master’s degree (128, 40.00%) or a bachelor’s degree (106, 33.13%). The rheumatologists mainly worked in tertiary hospitals (261, 81.56%). The median number of patients with SS treated per month was 15 (11, 50) and the median prevalence of patients with SS among all rheumatology patients was 6.66% (6–10%).

### Attitudes and perceptions of rheumatologists and patients towards TCM for SS

**Fig. 1:**
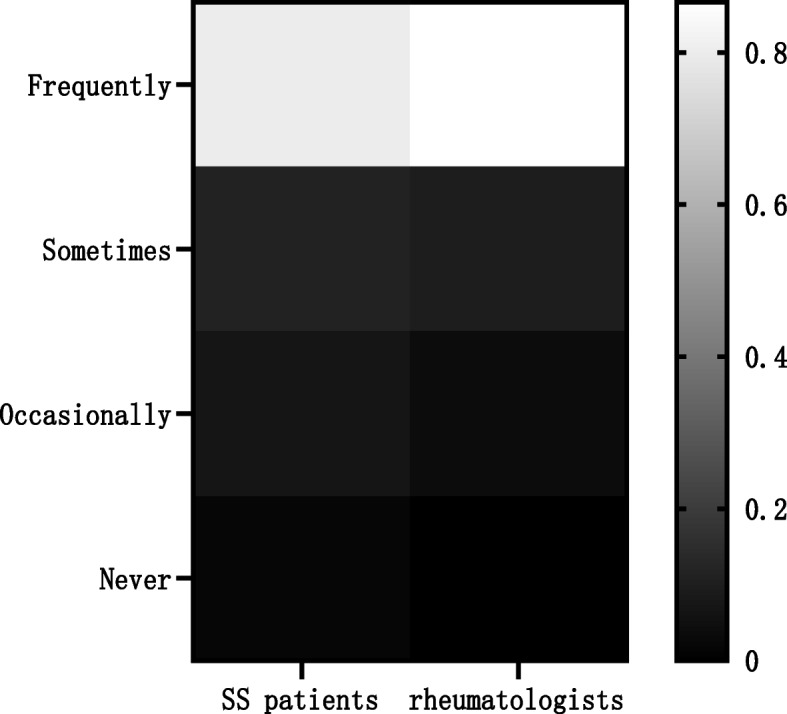
The proportion of rheumatologists recommending or SS patients receiving TCM. The vast majority of SS patients and Chinese rheumatologists expressed a willingness to accept or recommend TCM treatment, but rheumatologist recommendations were more common than patient acceptance. SS: Sjögren’s syndrome; TCM: traditional Chinese medicine

**Fig. 2:**
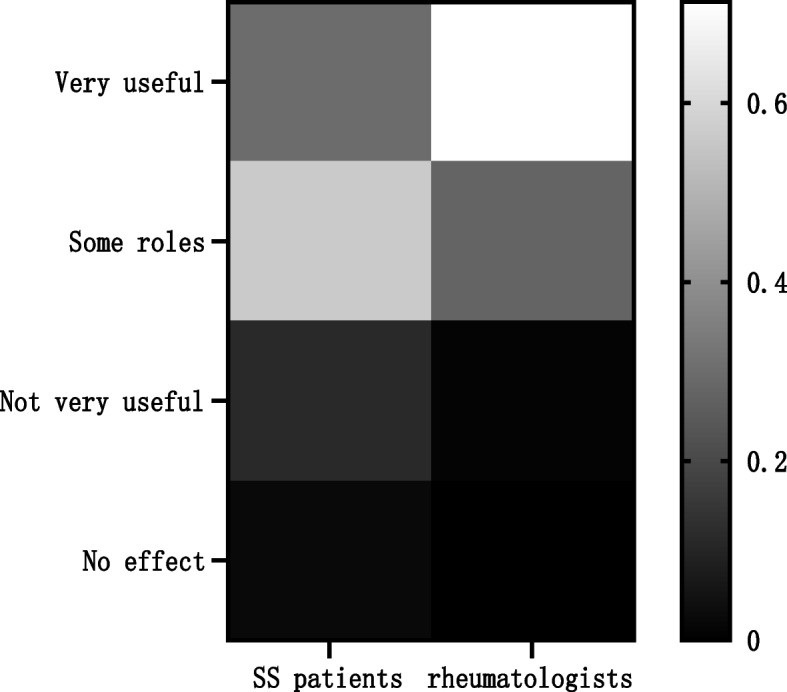
The proportion of rheumatologists or SS patients with different degrees of recognition of the efficacy of TCM.Chinese rheumatologists were more certain of the therapeutic effects of TCM, compared with the SS patients; the patients with SS mostly thought that TCM was somewhat useful, while the vast majority of rheumatologists thought TCM was very useful. SS: Sjögren’s syndrome; TCM: traditional Chinese medicine

The vast majority of SS patients and Chinese rheumatologists expressed a willingness to accept or recommend TCM treatment, but rheumatologist recommendations were more common than patient acceptance (*P* < 0.05). Chinese rheumatologists were more certain of the therapeutic effects of TCM, compared with the SS patients (*P* < 0.05); the patients with SS mostly thought that TCM was somewhat useful (531 cases, 56.37%), while the vast majority of rheumatologists thought TCM was very useful (228 cases, 71.25%, Table 1, Figs. 1, 2).

**Table 1:** Attitudes and perceptions of rheumatologists and SS patients towards TCM for SS

Project	SS patients (*n* = 942)	Rheumatologists (*n* = 320)	*P*-value
	**n (%)**	**n (%)**	
**Whether to recommend or receive TCM treatment**			
Frequently	753 (79.94)	277 (86.56)	0.00
Sometimes	108 (11.46)	31 (9.69)	
Occasionally	65 (6.90)	12 (3.75)	
Never	16 (1.70)	0 (0.00)	
**Therapeutic effect of TCM in the treatment of SS**			
Very useful	283 (30.04)	228 (71.25)	0.00
Somewhat useful	531 (56.37)	89 (27.81)	
Not very useful	106 (11.25)	3 (0.94)	
No effect	22 (2.34)	0 (0.00)	

Values are expressed as n (%). For each category, the total percentage = 100%. The number of evaluable SS patients was 942, and 320 Chinese rheumatologists participated in the survey.SS: Sjögren’s syndrome; TCM: traditional Chinese medicine

### Analysis of factors influencing the use of TCM treatments by rheumatologists and SS patients

“Whether or not they had received TCM treatment” was selected to represent the application of TCM by patients with SS. Results were analyzed via logistic regression. The patients’ use of TCM was a dichotomous variable, therefore binary logistic regression analysis was used to investigate the relationship between the response variable and the explanatory variables. In the dichotomous variable model, the response variable Y had two possible values (0 = no, 1 = yes), and the explanatory variables were gender, age, presence of medical insurance, annual household income, and duration of illness. Of these, age and duration of illness were statistically significant when included in the logistic regression analysis model. The Hosmer and Lemeshow test for goodness of fit was 1.000 (*P* > 0.05), suggesting that the model was a good fit and that dichotomous logistic regression analysis was applicable to these data.

Regression analysis showed that age and disease duration were the main factors that influenced patient use of TCM (*P* < 0.05). Controlling for other factors, the probability of patients applying TCM was positively correlated with age and disease duration. The probability of patients aged 41–50, 51–60, and ≥ 61 years utilizing TCM was 2.200, 2.217, and 2.388 times higher than that of patients ≤ 30 years old, respectively (*P* < 0.05). Furthermore, the probability of patients with a disease duration of 1–3 years, 4–10 years, 11–20 years, and ≥ 20 years utilizing TCM was 1.607, 2.636, 4.474, and 6.794 times more likely compared with patients with a disease duration of < 1 year, respectively (*P* < 0.05, Table 2).

**Table 2:** Factors influencing the utilization of TCM

Factor	Estimate	Wald	Sig	OR	95% CI
**Age (years)** ≤ 30					
31–40	0.316	0.122	0.727	1.117	(0.601, 2.073)
41–50	0.307	6.590	0.010	2.200	(1.205, 4.017)
51–60	0.300	7.030	0.008	2.217	(1.231, 3.993)
≥ 61	0.333	6.826	0.009	2.388	(1.243, 4.589)
**Course of disease (years)** < 1					
1–3	0.319	5.521	0.019	1.607	(1.082, 2.386)
4–10	0.312	23.894	0.000	2.636	(1.787, 3.888)
11–20	0.401	23.003	0.000	4.474	(2.426, 8.254)
≥ 21	1.074	8.749	0.003	6.794	(1.909, 24.192)
**Gender** male					
female	0.303	0.180	0.672	1.137	(0.628, 2.058)
**Presence of medical insurance** no					
general medical insurance	0.622	0.394	0.530	1.478	(0.436, 5.003)
commercial insurance	0.499	0.135	0.714	0.833	(0.303, 2.213)
other	0.778	1.475	0.225	0.389	(0.085, 1.785)
**Annual household income(¥:yuan)** < 100,000					
100,000–300,000	0.342	0.721	0.396	0.748	(0.383, 1.462)
300,000–500,000	0.717	2.621	0.105	3.192	(0.783, 13.012)
500,000–1,000,000	0.342	0.725	0.394	1.338	(0.685, 2.614)
≥ 1,000,000	0.323	0.003	0.954	1.019	(0.541, 1.919)

*Sig*. Significance, *OR* Odds ratio, *TCM* Traditional Chinese medicine

Our logistic regression showed that all rheumatologists would recommend TCM treatment, but the degree of that recommendation was different. The relationship between the response variable and the explanatory variables was examined using an ordered multicategorical logistic regression analysis because TCM treatment recommendation was a hierarchical variable. In the ordered variables model, the response variable Y took three possible values (1 = often would, 2 = sometimes would, and 3 = occasionally would), and the explanatory variables were gender, professional category, highest education, workplace, and years working in rheumatology. The *P* value of the parallel line test result was 0.712, which does not reject the original hypothesis; that is, the regression coefficients of the independent variables are equal, and the parallelism test is valid. The likelihood ratio test result was *P* < 0.05, indicating that the model was meaningful overall. The *P* value of the goodness-of-fit test result was 1.000 (*P* > 0.05), which can be considered a good fit.

Regression analysis showed that the number of years of rheumatology work experience and specialty type affected the degree of TCM treatment recommendation (*P* < 0.05). Controlling for other factors, the probability of not recommending TCM by rheumatologists with < 2 years and 2–5 years of rheumatology and immunology experience was 2.863 and 2.927 times higher than that of rheumatologists with > 15 years of experience, respectively (*P* < 0.05). For rheumatologists specializing in clinical medicine and Chinese medicine, the probability of them not recommending TCM was 2.886 (*P* < 0.05) and 0.629 (*P* > 0.05) times higher than that of rheumatologists who specialized in combined Chinese and Western medicine, respectively (Table 3).

**Table 3:** Factors influencing rheumatologist recommendation of TCM

Factor	Estimate	Wald	Sig	OR	95% CI
**Work experience (years)** > 15					
≤ 2	1.052	6.994	0.008	2.863	(0.272, 1.831)
2–5	1.074	11.182	0.001	2.927	(0.444, 1.703)
5–15	0.381	1.722	0.189	1.464	(-0.188, 0.950)
**Professional category** Combination of Chinese and Western Medicine					
Clinical Medicine	1.060	11.852	0.001	2.886	(0.457, 1.664)
Traditional Chinese Medicine	-0.464	2.555	0.110	0.629	(-1.033, 0.105)
**Gender** Female					
male	0.467	1.314	0.252	0.535	(-0.380, 1.451)
**Highest education** Other					
Bachelor degree	2.156	2.357	0.125	-3.310	(-7.535, 0.916)
Master’s degree	2.232	0.865	0.352	-2.077	(-6.452, 2.299)
Doctoral degree	2.250	0.479	0.489	-1.558	(-5.967, 2.852)
**Workplace** Hospitals below Grade A					
Third-class first-class hospital	1.040	2.918	0.566	19.256	(17.218, 21.295)
Tertiary hospital	1.142	1.963	0.479	20.486	(18.249, 22.724)
Second-class hospital	1.367	1.240	0.672	19.193	(10.831, 22.308)

*Sig*. Significance, *OR* Odds ratio, *TCM* Traditional Chinese medicine

### Perceived advantages and disadvantages of TCM by SS patients and rheumatologists

**Fig. 3:**
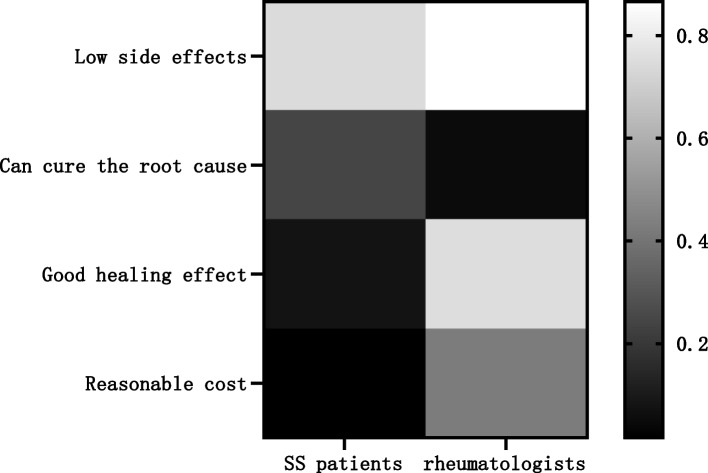
The proportion of rheumatologists or SS patients choosing different disadvantages of TCM.Patients perceived the main advantages of TCM as having fewer side effectsand being able to cure the disease, while rheumatologists perceived the main advantages of TCM as having fewer side effects and good efficacy. SS: Sjögren’s syndrome; TCM: traditional Chinese medicine

**Fig. 4:**
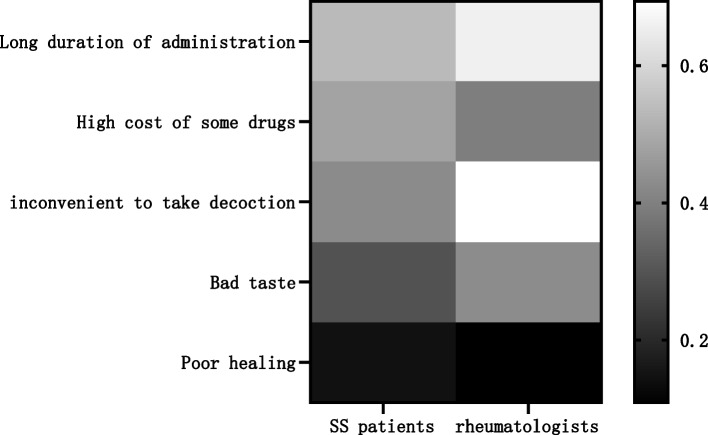
The proportion of rheumatologists or SS patients choosing different disadvantages of TCM. Patients perceived the main disadvantages of TCM as taking a long time, high cost, and inconvenient decoction, while the main disadvantages of TCM as perceived by rheumatologists were inconvenient decoction, long duration of administration, and bad taste. SS: Sjögren’s syndrome; TCM: traditional Chinese medicine

There were significant differences in the perceived advantages and disadvantages of TCM for SS reported by rheumatologists and patients (*P* < 0.05). Patients perceived the main advantages of TCM as having fewer side effects (700 cases, 74.31%) and being able to cure the disease (593 cases, 62.95%), while rheumatologists perceived the main advantages of TCM as having fewer side effects (277 cases, 86.56%) and good efficacy (240 cases, 75.00%). Patients perceived the main disadvantages of TCM as taking a long time (503 cases, 53.40%), high cost (453 cases, 48.09%), and inconvenient decoction (403 cases, 42.78%), while the main disadvantages of TCM as perceived by rheumatologists were inconvenient decoction (222 cases, 69.38%), long duration of administration (211 cases, 65.94%), and bad taste (137 cases, 42.81%) (Table 4 and Figs. 3, 4).

**Table 4:** Advantages and disadvantages of TCM from rheumatologist and SS patient perspectives

Project	SS patients (*n* = 942)	rheumatologists (*n* = 320)	*P*-value
	**n (%)**	**n (%)**	
**Advantages of Chinese Medicine**			
Low side effects	700 (74.31)	277 (86.56)	0.00
Can cure the root cause	593 (62.95)	17 (5.31)	
Good healing effect	73 (7.75)	240 (75.00)	
Reasonable cost	16 (1.70)	137 (42.81)	
**Disadvantages of Chinese medicine**			
Long duration of administration	503 (53.40)	211 (65.94)	0.00
High cost of some drugs	453 (48.09)	127 (39.69)	
Inconvenient to take decoction	403 (42.78)	222 (69.38)	
Bad taste	280 (29.72)	137 (42.81)	
Poor healing	138 (14.65)	35 (10.94)	

The numerical values are expressed as n (%). For each category, the total percentage can be > 100%, as multiple responses were allowed. The number of evaluable participants was 942 SS patients and 320 Chinese rheumatologists. SS: Sjögren’s syndrome; TCM: traditional Chinese medicine

### Recommended or expected therapies

**Fig. 5:**
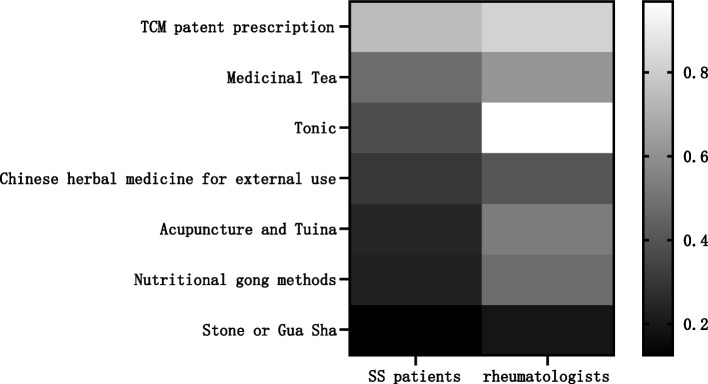
The proportion of rheumatologists and SS patients who recommend or expect different treatment methods in TCM. There was a significant difference between the Chinese medicine therapies expected by patients and recommended by rheumatologists. SS: Sjögren’s syndrome; TCM: traditional Chinese medicine

There was a significant difference between the Chinese medicine therapies expected by patients and recommended by rheumatologists (*P* < 0.05). The most expected TCM therapies by patients were, in order, TCM patent prescriptions (711 cases, 75.48%), herbal tea (462 cases, 49.04%), tonics(366 cases, 38.85%), and topical Chinese medicine (294 cases, 31.21%). The most recommended TCM therapies by rheumatologists were, in order, tonics (310 cases, 96.88%), TCM patent prescriptions (262 cases, 81.88%), herbal tea (197 cases, 61.56%), and acupuncture and massage (171 cases, 53.44%) (Table 5 and Fig. 5).

**Table 5:** Recommended or expected TCM treatment options

Project	SS patients (*n* = 942)	Rheumatologists (*n* = 320)	*P*-value
	**n (%)**	**n (%)**	
**Recommended/expected TCM treatment options**			
TCM patent prescription	711 (75.48)	262 (81.88)	0.00
Medicinal Tea	462 (49.04)	197 (61.56)	
Tonic	366 (38.85)	310 (96.88)	
Chinese herbal medicine for external use	294 (31.21)	130 (40.63)	
Acupuncture and Tuina	235 (24.95)	171 (53.44)	
Nutritional gong methods	222 (23.57)	154 (48.13)	
Stone or Gua Sha	120 (12.74)	62 (19.38)	

The numerical values are expressed as n (%). For each category, the total percentage is > 100%, as multiple responses were allowed. The number of evaluable participants was 942 SS patients and 320 Chinese rheumatologists

*SS* Sjögren’s syndrome, *TCM* Traditional Chinese medicine

## Discussion

### The survey population covers a wide range of people, the results have some generalizable value in China

This survey covered over 30 provinces and cities across China, with a male-to-female ratio of approximately 1:14, the largest proportion of patients were aged 51–60 years (33.70%) and had a disease duration of 4–10 years (40.00%), which is consistent with the epidemiological data described in previous studies [24–27]. The Chinese rheumatologists who participated in the study covered 30 provinces and cities across China, with 65.94% holding master’s degrees. Participating rheumatologists were predominantly from tertiary referral hospitals (81.56%) and had a median work experience of 12 (6, 15) years, a relatively high education level and work hospital level, and a relatively rich clinical experience. The median number of SS patients treated by rheumatologists per month was 15, accounting for a median proportion of 6.66% (6–10%) of all rheumatic patients, which is congruent with epidemiological reports [28–31]. These results suggest that the data from this survey have some generalizable value in China.

### TCM was accepted by SS patients and rheumatologists, especially older patients and rheumatologists with a longer working experience

The life experiences and wisdom of the elders among patients and rheumatologists may permit the appreciation and comprehension of the philosophy of life embedded in TCM. Our survey found that patients and rheumatologists generally had positive and accepting attitudes toward TCM for SS, expecting TCM to effectively relieve symptoms and treat the disease. Patients with SS were more receptive to TCM compared with patients with other chronic diseases [32], which might be related to the fact that there are currently no reliable Western treatment protocols for SS. Correspondingly, patients who received TCM were predominantly of advanced age and had prolonged illnesses, and the likelihood of a rheumatologist recommending TCM was directly proportional to their years of practice, with a particularly high likelihood among those trained in Chinese medicine and combined Chinese and Western medicine. We hypothesize that this finding is because older patients with prolonged illnesses are more likely to have various comorbidities and multi-system damage [33–36], negatively impacting their physical and mental states and making them more eager to seek help from multiple sources. Furthermore, rheumatologists with a TCM background or longer working years tend to be more experienced, have more knowledge of TCM, understand the current treatment bottlenecks for SS, and do not refuse any kind of treatment modality. Older patients and older rheumatologists may also be more conservative in their thinking and thus more inclined to accept traditional treatments. Moreover, as they have accumulated more life experiences and wisdom, these individuals may be better able to understand and appreciate the philosophy of life contained in TCM.

### Long treatment periods are not synonymous with TCM therapy. The symptoms and the root cause can both be treated by TCM and western medicine

This survey found that more than 60% of patients were worried about the “long medication cycle” of TCM and more than 40% expected TCM to “cure the root cause”, reflecting their limited knowledge of SS and their solidified thinking about TCM. SS is a chronic autoimmune disease. The main clinical goal of SS treatments is to control symptoms and delay disease progression. Current conventional treatment drugs such as leflunomide and methotrexate have an effective cycle of 3–6 months [37, 38]. TCM can also yield effective treatment within 3 months through accurate syndrome differentiation [9–11, 39]. Since the nature of the disease determines the long treatment cycle, which is unrelated to the treatment method, the long treatment is not equivalent to TCM treatment.

Immune imbalance is the root cause of autoimmune diseases. Rregulating immune function and restoring the balance between different types of lymphocytes is the treatment for the underlying cause, while simply relieving symptoms is treating the surface problem. Artificial tears, artificial saliva, and non-steroidal anti-inflammatory drugs can alleviate symptoms such as dryness and pain, but they do not target the pathogenesis of SS itself. Hydroxychloroquine, leflunomide, and bio-agents, which regulate immune balance [40], treat the root cause of SS, rejecting the patients’ claim. Some clinicians believe that TCM is only effective at relieving symptoms, but there are reports that TCM can regulate the balance of immune cells and restore immune homeostasis [12, 41]. The symptoms and the root cause are therefore not unique to TCM or Western medicine, suggesting that Chinese and Western medicine are not yet fully understood and that communication between rheumatologists and patients should be strengthened.

### Convenience and efficiency needs of rheumatologists and SS patients suggests that future research and development should focus on TCM patent prescriptions with clear ingredients and mechanisms

This survey found that a unique advantage of Chinese medicine recognized by both rheumatologists and SS patients was its favorable side effect profile. However, the two participant groups differed in their choice of therapy. More than 40% of patients said inconvenient decoction was a major disadvantage of TCM, were more concerned about the convenience and taste of the medication, and preferred dosage forms such as TCM patent prescriptions and tea drink substitutes. A long treatment duration is seen as a problem with treatment for SS by many patients, who hope to simplify future treatments and reduce the pain of taking medication. Improvements in TCM dosage form are required to encourage treatment adoption. TCM may also be more easily accepted by rheumatologists and SS patients if its efficacy is guaranteed. TCM patent prescriptions, which play an important role in modern TCM treatments, not only have obvious efficacy advantages but also meet the needs of most patients [42, 43]. Widely used medicine ingredients include plant extracts such as Leigongjiang polysaccharide and Baishaoside [44], and herbal oral rinses have been effective at improving the dry mouth symptoms of patients with SS [45], supporting the feasibility of the idea of balancing efficacy and convenience in TCM. Convenient and efficient TCM patent prescriptions are a major requirement for rheumatologists and patients, which suggests that future work should focus on developing TCM patent prescriptions with clear ingredients and mechanisms to safeguard efficacy.

## Conclusion and limitation

**Fig. 6:**
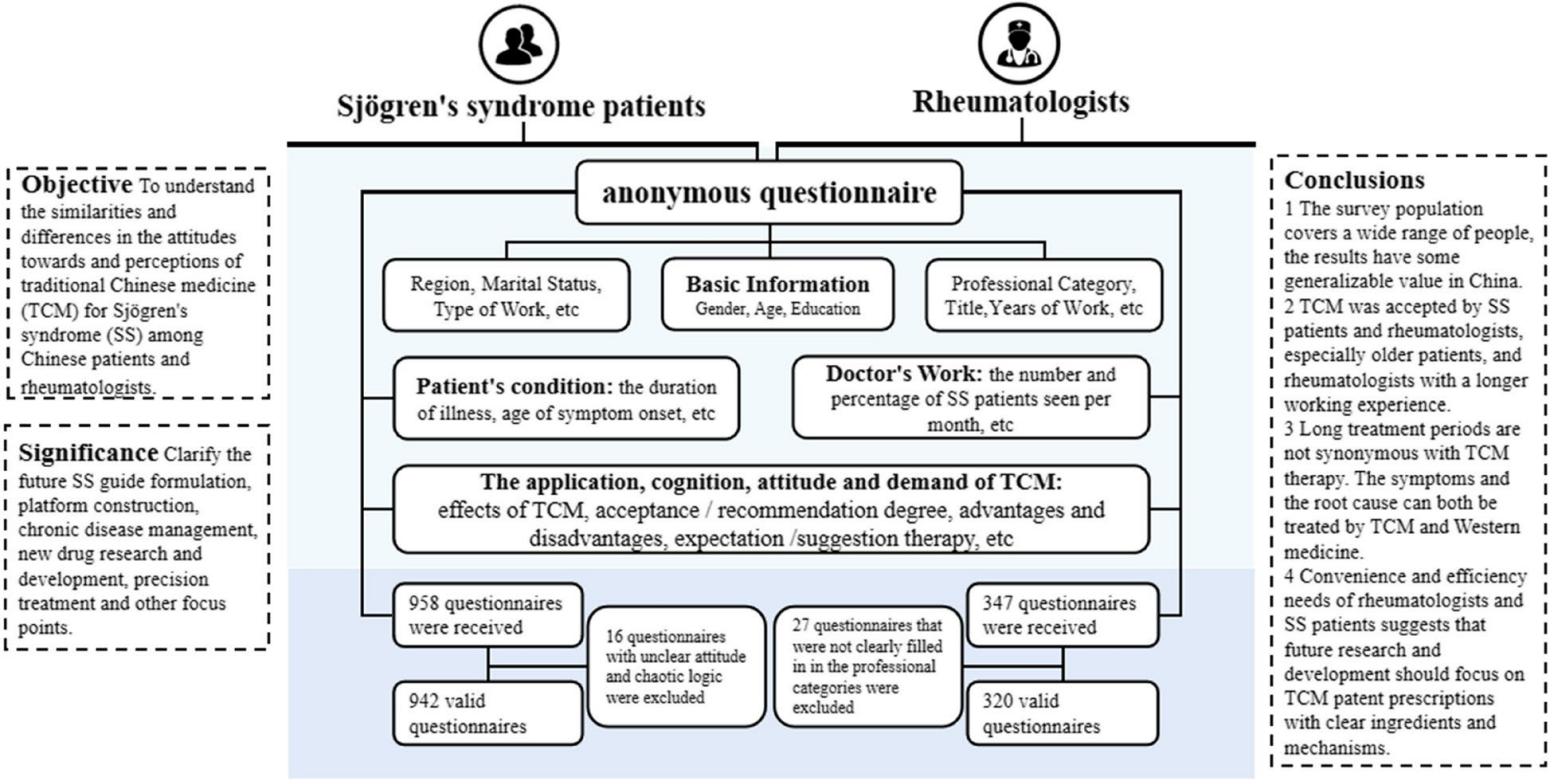
Design thinking diagram. SS: Sjögren’s syndrome; TCM: traditional Chinese medicine

This study found that TCM therapies have been widely experienced by many SS patients, who view them as advantageous, and they are also highly regarded by Chinese rheumatologists. However, our poor understanding of SS hinders the development of improved TCM therapies. Furthermore, the contradiction between the dosage form and efficacy of TCM is a major problem in this field. Data from this survey emphasizes the demand of SS patients for convenient and efficient TCM formulations, suggesting that the development of TCM patent prescriptions with clear compositions and mechanisms should be prioritized (Fig. 6).

Although this survey can reflect the attitudes and perceptions of SS patients and Chinese rheumatologists towards TCM to some extent, this work does have several limitations. First, more than 70% of the surveyed patients were diagnosed at Western medical hospitals, and the selection bias of the initial treating hospital must be considered. Second, more than 70% of the surveyed rheumatologists have a background in Chinese medicine, and the uneven ratio of specialties may also contribute to bias. Furthermore, there are other problems such as the relatively limited number of survey respondents and an uneven regional distribution. Future works must include a large sample through a multicenter survey and should seek to accurately understand the clinical needs of TCM, explore new ideas for TCM development, and strive to effectively guide clinical treatment.

## Data Availability

The datasets used and/or analyzed during the current study are available from the corresponding author on reasonable request.

## Abbreviation

TCM: Traditional Chinese medicine
SS: Sjögren's syndrome
